# RECQ helicases are deregulated in hematological malignancies in association with a prognostic value

**DOI:** 10.1186/s40364-016-0057-4

**Published:** 2016-02-13

**Authors:** Elena Viziteu, Alboukadel Kassambara, Philippe Pasero, Bernard Klein, Jerome Moreaux

**Affiliations:** Laboratory for Monitoring Innovative Therapies, Department of Biological Hematology, Hôpital Saint-Eloi - CHRU de Montpellier, 80, av. Augustin Fliche, 34295 Montpellier, Cedex 5 France; Institute of Human Genetics, CNRS-UPR1142, Montpellier, F-34396 France; University of Montpellier 1, UFR de Médecine, Montpellier, France

**Keywords:** RECQ helicases, Gene expression, Hematological malignancies, Prognostic markers, Therapeutic targets

## Abstract

**Background:**

RECQ helicase family members act as guardians of the genome to assure proper DNA metabolism in response to genotoxic stress. Hematological malignancies are characterized by genomic instability that is possibly related to underlying defects in DNA repair of genomic stability maintenance.

**Methods:**

We have investigated the expression of *RECQ* helicases in different hematological malignancies and in their normal counterparts using publicly available gene expression data. Furthermore, we explored whether *RECQ* helicases expression could be associated with tumor progression and prognosis.

**Results:**

Expression of at least one *RECQ* helicase family member was found significantly deregulated in all hematological malignancies investigated when compared to their normal counterparts. In addition, *RECQ* helicase expression was associated with a prognostic value in acute myeloid leukemia, chronic lymphocytic leukemia, lymphoma and multiple myeloma.

**Conclusion:**

*RECQ* helicase expression is deregulated in hematological malignancies compared to their normal counterparts in association with a prognostic value. Deregulation of RECQ helicases appears to play a role in tumorigenesis and represent potent therapeutic targets for synthetic lethal approaches in hematological malignancies.

**Electronic supplementary material:**

The online version of this article (doi:10.1186/s40364-016-0057-4) contains supplementary material, which is available to authorized users.

## Background

The RECQ family of DNA helicases is a family of conserved enzymes that display highly-specialized and vital roles in the maintenance of genome stability [1].

In humans, RECQ helicase family has five members with similar catalytic core: RECQ1, BLM, WRN, RECQ4 and RECQ5 [1]. Mutations in three of the five human RECQ helicases, BLM, WRN and RECQ4, lead to genetic disorders as Bloom, Rothmund-Thompson and Werner’s syndromes that are associated with cancer predisposition, premature ageing and developmental abnormalities [1, 2].

The Bloom’s syndrome helicase, BLM has DNA annealing and unwinding activities. Through its interaction with TOPOIIIα, BLM unwinds the short stretches of naked duplex DNA and processes homologous recombination (HR) intermediates containing a double holiday junction [1, 2]. This helicase appears to prefer specific structures including D-loops and Holliday junctions and promotes Holliday junction branch migration [3]. It may suppress hyper-sister chromatid exchange (SCE) by disruption of D-loop recombination intermediates and also might be involved in the suppression of crossing over during homology-mediated recombination [4]. BLM-mediated crossover suppression may involve synthesis-dependent strand annealing (SDSA) [3]. This helicase facilitates telomere replication by resolving G4 structures [5]. Defects in BLM are also associated with the cancer phenotype [4].

Unlike the other members of the RECQ helicase family, the Werner’s syndrome helicase, WRN contains both the classical helicase activity and 3’-to 5’ exonuclease activity that target multiple DNA or RNA-DNA hybrid structures [1, 2]. As BLM, WRN appears to have robust in vitro G4 unwinding activity [6] and plays a specialized role in telomere replication by disruption of G-quadruplex stretches [7]. Another specific substrate of WRN is D-loop. WRN repress the inappropriate telomeric recombination intermediates through its ability to resolve the D-loops [8]. This helicase was found to be involved in the repair of double strand DNA breaks and studies on Werner’s syndrome fibroblasts have shown defects in recombination intermediate resolution which suggests that WRN is involved in HR [9]. WRN can bind to NBS1 [10], a member of the MRN complex, and to the Ku70/Ku80 heterodimer [11], a core non-homologous end joining DNA repair (NHEJ) complex.

RECQ1 is the shortest of the human RECQ family helicases. RECQ1 displays specific functions in branch migration and restart of reversed DNA replication forks upon DNA topoisomerase I inhibition that is not shared by other human RECQ helicases [12, 13]. Studies have also shown that RECQ1 plays a role in DNA strand breaks repair, mismatch repair and resistance to replication stress [1, 2, 13, 14]. RECQ1 can also contribute to tumor development and progression by regulating the expression of key genes that promote cancer cell migration, invasion and metastasis [15].

The gene encoding for RECQ5 helicase was found to be localized on human chromosome 17q23–25, a region associated with both breast and ovarian cancer [16]. RECQ5 was found to cause a significant increase in the frequency of spontaneous SCE [1, 2]. As BLM, RECQ5 was shown to play an essential role in suppression of crossovers [17]. RECQ5 was identified as a potential proto-oncogene in mouse leukemia [18]. RECQ5 is the only member of RECQ family associated with RNA polymerase II, maintaining genomic stability during transcription [19].

RECQ helicase are at the crossroad between replication, recombination, DNA repair and transcription and could represent potent therapeutic targets for cancer therapy [20]. Hematological malignancies are characterized by genomic instability that is possibly related to underlying defects in DNA repair of genomic stability maintenance. Since these helicases play important roles in the maintenance of chromosomal stability [21], we focused on *RECQ* helicases expression in hematological cancers compared to their normal counterparts and the association with prognostic impact.

## Results and discussion

**Fig. 1:**
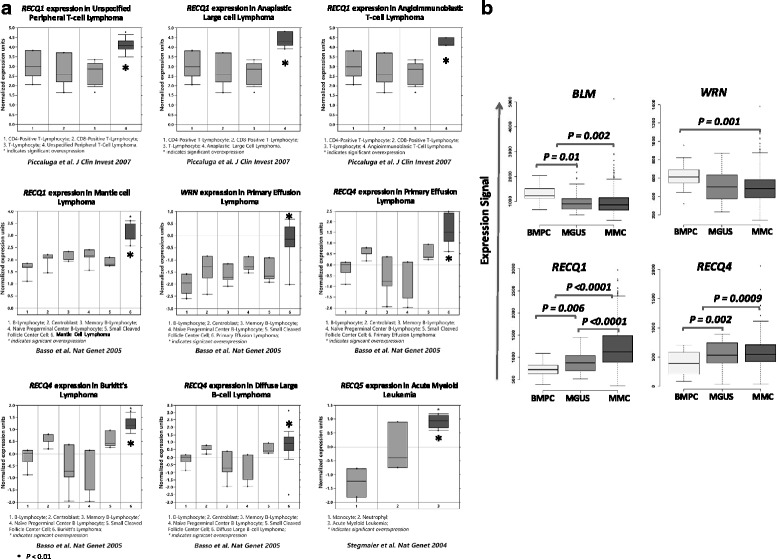
Increased RECQ helicase gene expression in hematological malignancies compared to normal counterparts using Oncomine database (**a**) and Genomicscape database (**b**). Data sets in a given panel were from the same study. GEP data are log transformed (Oncomine) or not (Genomicscape) and normalized as previously described [64]

*RECQ* helicase gene expression levels were analyzed in different types of hematological malignancies and in their normal counterparts using Oncomine Cancer Microarray database [22] as indicated in Table 1. Abnormal expression of at least one *RECQ* helicase was identified in all analyzed hematological malignancies (Table 1). *RECQ1* was found to be significantly overexpressed in mantle cell lymphoma (*P* = 0.0025) [23], in unspecified peripheral T-cell lymphoma (*P* = 0.0038) [24], anaplastic large lymphoma (*P* = 0.0024) [24] and angioimmunoblastic large cell lymphoma (P = 0.001) [24] (Fig. 1a).

**Table 1:** *RECQ* family member expression in hematological malignancies compared to that of their normal tissue counterparts using publicly available gene expression data, including the Oncomine Cancer Microarray and Genomicscape databases

	Hematological Malignancies	Datasets	Gene overexpression compared to normal tissue counterpart				
			*RECQ1*	*BLM*	*WRN*	*RECQ4*	*RECQ5*
Myeloid Neoplasms	Acute Myeloid Leukemia	12	No	No	No	No	YES
B-cell neoplasms	Primary Effusion Lymphoma	8	No	No	YES	YES	No
	Chronic Lymphocyte Leukemia	11	No	No	YES	No	No
	Diffuse Large B-cell Lymphoma	8	No	No	No	YES	No
	Burkitt's Lymphoma	8	No	No	No	YES	No
	Mantle Cell Lymphoma	11	YES	No	No	No	No
	Multiple Myeloma	1	YES	YES	YES	YES	No
T-cell neoplasms	Unspecified Peripheral T-cell Lymphoma	11	YES	No	No	No	No
	Anaplastic Large Cell Lymphoma	11	YES	No	No	No	No
	Angioimmunoblastic T-Cell Lymphoma	11	YES	No	No	No	No

*WRN* is significantly overexpressed in primary effusion lymphoma compared to normal B cells (*p* = 0.003) [23] (Fig. 1a).

*RECQ4* expression was increased in Burkitt Lymphoma (*P* = 0.001) [23, 25], in diffuse large B cell lymphoma (*P* = 0.004) [23] and also in primary effusion lymphoma (*P* = 0.005) [23] compared to normal counterpart (Fig. 1a).

*RECQ5* is significantly overexpressed in acute myeloid leukemia (*P* = 0,001) [26] (Fig. 1a).

Comparing *RECQ* helicases expression between normal plasma cells (BMPC), premalignant cells from MGUS patients and multiple myeloma cells (MMC) [27], *BLM* and *WRN* were found to be significantly downregulated in MMC compared to normal BMPC (*P* = 0.002 and *P* = 0.001 respectively) (Fig. 1b). A decreased expression of *BLM* was also observed in MGUS compared to BMPC (*P* = 0.01). *RECQ1* and *RECQ4* are overexpressed in MGUS (*P* = 0.006 and *P* = 0.002) and MMC (*P* < 0.0001 and *P* = 0.0009) compared to BMPC. Furthermore, a significant increased expression of *RECQ1* in MMC compared to MGUS was identified (Fig. 1b).

Furthermore, using the human protein atlas database [28–30], the expression of RECQ1, RECQ4 and RECQ5 could be confirmed at protein level in myeloid and lymphoid cancer cell lines (Additional file 1: Figure S1).

We investigated whether *RECQ* helicases expression could be associated with tumor progression and prognosis in hematological malignancies (Table 2).

**Table 2:** Link between *RECQ* helicase gene expression and prognostic value in hematological malignancies

RECQ helicase	Prognostic value					
	AML		LLC	FL	DBLCL	MM
	normal karyotype	abnormal karyotype				
*WRN*	-	-	GOOD	GOOD	-	BAD
*BLM*	BAD	GOOD	-	-	-	-
*RECQ1*	BAD	-	-	BAD	-	BAD
*RECQ4*	GOOD	GOOD	-	GOOD	-	BAD
*RECQ5*	BAD	-	BAD	BAD	GOOD	-

GOOD : A high *RECQ* helicase expression is associated with a better outcome

BAD : A high *RECQ* helicase expression is associated with a poor prognosis

**Fig. 2:**
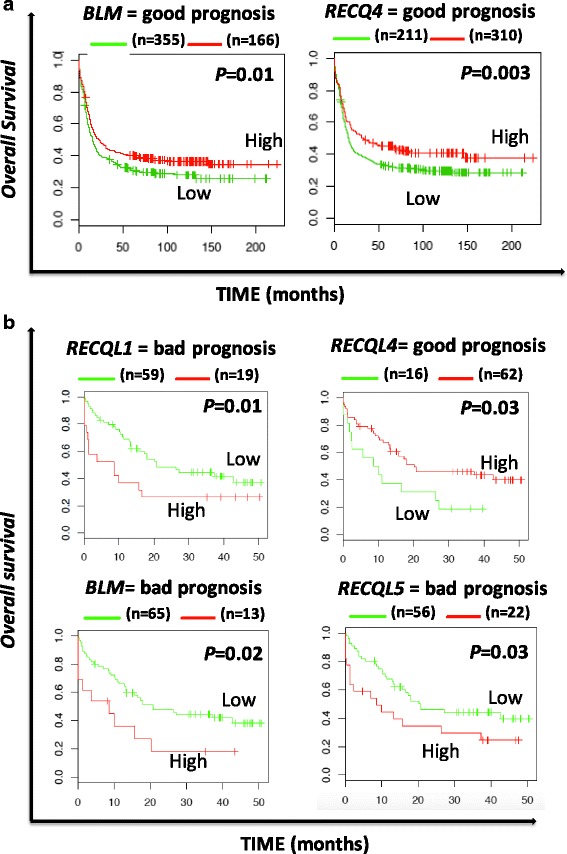
Overall survival related to RECQ helicase gene expression in acute myeloid leukemia with abnormal karyotype (**a**) and normal karyotype (**b**). The prognostic value of gene expression was determined using the MaxStat R function in R software. The overall survival of subgroups of patients was compared with the log-rank test and survival curves computed with the Kaplan–Meier method (R software) [66]

In AML patients with abnormal karyotype (Verhaak cohort, *N* = 521 patients) [31], a high expression of *BLM* and *RECQ4* is associated with a better overall survival (OS) (*P* = 0.01 and *P* = 0.003). At the opposite, high *RECQ5* expression was linked with a poor prognosis in the same cohort of patient (*P* = 0.008) (Fig. 2a). In cox multivariate analysis, only *RECQ5* expression kept prognostic value (*P* = 0.01, hazard ratio (HR) = 1.43).

In AML with normal karyotype, (Metzeler cohort, *N* = 78) [32], gene expression of four *RECQ* helicases out of five were identified to predict for OS. High expression of *RECQ1* (*P* = 0.02), *BLM* (*P* = 0.01) and *RECQ5* (*P* = 0.03) were found to be associated with poor prognosis. In contrast, high *RECQ4* expression was linked with a better outcome (*P* = 0.03) (Fig. 2b). When tested together in a cox multivariate analysis, only *RECQ4* expression remained significant (*P* = 0.009; HR = 0.4).

Interestingly, *RECQ5* overexpression was only identified in myeloid malignancies in association with an adverse prognosis. *RECQ5* increased expression was recently reported in JAK2V617F myeloproliferative neoplasms [33]. RECQ5 depletion in JAK2V617F-mutant cells impairs replication after hydroxyurea treatment leading to a significant increased double-stranded breaks and apoptosis [33]. RECQ5 represents a potent regulator of genome stability in myeloproliferative neoplasms in association with drug resistance [33]. *RECQ5* overexpression could also be involved in AML pathophysiology and chemoresistance.

Even if RECQ1 mutations have been recently shown to been associated with predisposition to breast cancer [34, 35], no link between *RECQ1, BLM, WRN*, and *RECQ4* deregulation and lymphoid malignancies were previously reported.

**Fig. 3:**
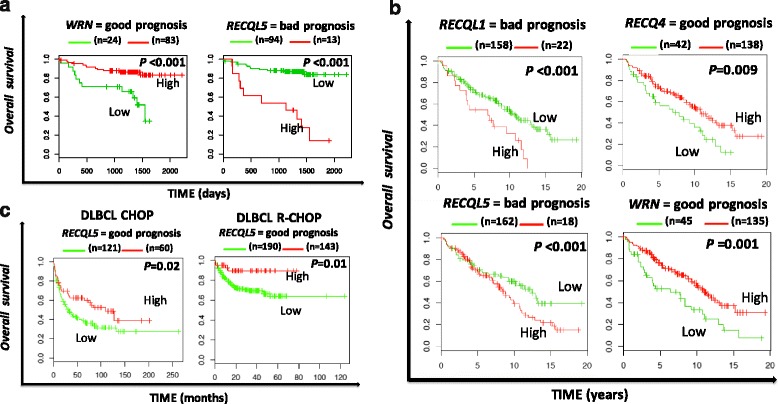
Overall survival related to *RECQ* helicase gene expression in chronic lymphocytic leukemia (**a**), follicular lymphoma (**b**) and diffuse large B cell lymphoma (**c**)

In chronic lymphocytic leukemia (CLL), a poor prognosis was linked with high *RECQ5* expression (*P* = 8E-8) and a better outcome was associated with high *WRN* expression (*P* = 0.0006) (Fig. 3a).

In a cohort of patients with follicular lymphoma (FL) (Staudt cohort, *N* = 180) [36], high *RECQ1* and *RECQ5* expression represented adverse prognostic factors (*P* = 0.003 and *P* = 0.0006 respectively) whereas *RECQ4* expression was found to be associated with a good prognosis (*P* = 0.009) (Fig. 3b). Interestingly, *RECQ1*, *RECQ4* and *RECQ5* expresison remained independent when tested in cox multivariate analysis (*P* < 0.0001; HR = 2.8; *P* = 0.002; HR = 0.49 and *P* < 0.01; HR = 1.78 respectively).

In diffuse large B cell lymphoma (DLBCL), only *RECQ5* expression was associated with a prognostic value. Low *RECQ5* expression was a poor prognostic marker in two independent cohorts of patients (*P* = 0.02 in a cohort of patients treated by combination of cyclophosphamide, doxorubicin, vincristine and prednisone (CHOP) therapy (*N* = 181) and *P* = 0.01 in a cohort of patients treated by Rituximab combined with CHOP (R-CHOP) regimen (*N* = 233)) (Fig. 3c) [37].

**Fig. 4:**
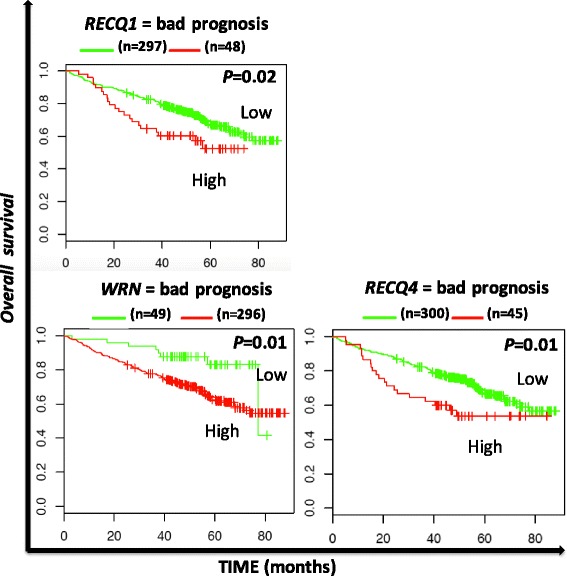
Overall survival related to *RECQ* helicase gene expression in multiple myeloma

In MM, high *RECQ1*, *WRN* and *RECQ4* expression are associated with an adverse prognosis in the UAMS cohort treated with total therapy 2 (Fig. 4) [27].

These data demonstrate a link between *RECQ* helicase expression and a prognostic value in different hematological malignancies.

Hematological malignancies are characterized by genomic instability that could be related to defects in DNA repair [21]. The RECQ family of DNA helicases is a family of conserved enzymes that display highly specialized and vital roles in the maintenance of genome stability. Mutations in three of the five human RECQ helicases, BLM, WRN and RECQL4, are associated with genetic disorders characterized by chromosomal instability and increased susceptibility to cancer including leukemia [38, 39]. Mutations in *BLM* result in a dramatic lowering of *BLM* mRNA levels and premature termination of protein translation owing to nonsense-mediated mRNA decay [1, 40]. Patients with Bloom syndrome exhibit cancer predisposition including most types of cancers and particularly non-Hodgkin’s lymphoma, leukemias and carcinomas of skin, breast and colon [1]. Interestingly, a low *BLM* expression is associated with a poor prognosis in AML with complex karyotypes (Fig. 2a). The cancer spectrum observed in patients with Werner’s syndrome is characterized mainly by cancers of mesenchymal origin and some epithelial cancers [1, 41]. RECQ4 mutations are found in Rothmund-Thomson syndrome, RAPADILINO syndrome and Baller-Gerold syndrome [1, 41]. Rothmund-Thomson syndrome are characterized by predisposition to mainly osteosarcoma whereas RAPADILINO syndrome are linked with lymphoma and osteosarcoma predisposition [1].

Specific recurrent chromosomal translocations have been associated with DNA repair deficiencies linked with repression of DDR (DNA damage response) genes in AML [42]. In PML-RARA, PML and BLM are delocalized from the nuclear bodies into microspeckled nuclear regions [43]. All trans retinoic acid (ATRA) treatment of APL patients leads to degradation of PML-RARA and relocalization of BLM to nuclear bodies [43] suggesting that PML-RARA are involved in genomic instability in APL through disruption of BLM and PML localization and activity. Interestingly, we reported that low *BLM* and *RECQ4* expression are associated with a poor prognosis in AML with abnormal karyotype (Fig. 2a), suggesting that downregulation of RECQ helicases could be involved in leukemogenesis and genomic instability. At the opposite, in AML with normal karyotype, *RECQ1*, *BLM* and *RECQ5* high expression are associated with a poor prognosis (Fig. 2b). As reported in solid cancer, *RECQ* helicase overexpression could be a marker of chemoresistance and higher proliferation helping AML cells to deal with replication stress [44, 45]. B lymphocytes are continuously produced during adult life and they undergo different genetic alterations associated with DNA breaks, including VDJ recombination, Ig class switch recombination and somatic hypermutation. These mechanisms must be tightly regulated to prevent tumorigenesis and ensure efficient immune response [46]. Collapsed DNA replication forks occurring in rapidly dividing lymphocytes leads to a restart failure and results in an interruption of the normal developmental program [47]. HR is required for lymphoid development [47]. Aberrations affecting HR actors are correlated with genomic instability in B cell cancers [48]. By their involvement in HR and also by their ability to resolve and to continue the normal fork replication after DNA damage or replication fork arrest, WRN [49], BLM [4], RECQ1 [12] and RECQ5 [50] helicases might be crucial in lymphoid development and aberration in their expression or function can lead to cancer genesis. Interestingly, low expression of WRN in CLL and FL, low *RECQ4* expression in FL and low *RECQ5* in DLBCL are associated with a poor prognosis (Fig. 3). Furthermore, high *RECQ5* expression in CLL and FL and high *RECQ1* expression in FL are associated with a poor prognosis and could be involved in chemoresistance. In lymphoma, deregulation of DDR is associated with tumorigenesis [51, 52], poor prognosis [53, 54] and could represent a potent therapeutic target [53, 55, 56].

In MM, patients with extensive chromosomal instability and replicative stress are associated with an adverse outcome [27, 57–59]. Accordingly, high *RECQ1*, *WRN* and *RECQ4* expression is associated with a significant poor survival in MM patients (Fig. 4). Although *WRN* was found to be significantly downregulated in MMC compared to normal BMPC (Fig. 1b), patients with high expression display a poor prognosis. *WRN* is located on chromosome 8p deleted in 25 % of MM patients without prognostic value [60]. These data could explain the significant downregulation of *WRN* expression in MM compared to normal BMPC.

Recently, a set of molecule inhibitors of WRN and BLM was characterized [61, 62]. These new molecules could open up new therapeutic strategies for targeting hematological malignancies characterized by RECQ helicase deregulation and a poor prognosis.

## Conclusion

The analysis reported here demonstrates that *RECQ* helicase expression is deregulated in hematological malignancies compared to their normal counterparts in association with a prognostic value in AML, CLL, lymphoma and MM. Deregulation of RECQ helicases appears to play a role in tumorigenesis and could be involved in genomic instability and chemoresistance in hematological malignancies. RECQ helicases represent potent therapeutic targets for synthetic lethal approaches.

## Methods

Databases: We used Oncomine Cancer Microarray database (http://www.oncomine.org) [22] and Genomicscape (http://genomicscape.com/) [63] to study gene expression of RECQ family members in nine different human hematological malignancies and their normal tissue counterpart as indicated in Table 1. To compare the gene expression of a tumor type to its normal counterpart, we used gene expression data from a same study with the same methodology. All data were log transformed, median centered per array, and the standard deviation was normalized to one per array [22].

Statistical comparisons were done with Mann–Whitney or Student *t*-test as previously published [64].

Prognosis values of each member of RECQ family in hematological malignancies were determined by using Maxstat R package based on publicly available data (Gene Expression Omnibus (http://www.ncbi.nlm.nih.gov/geo/); accession numbers GSE6891, GSE12417, GSE22762, GSE16131, GSE10846 and GSE4581) analyzed with Genomicscape [63] as previously reported [65].

## Additional file

Additional file 1: Figure S1. RECQ helicase gene and protein expression in myeloid and lymphoid cell lines using using the human protein atlas database. RECQ1, RECQ4 and RECQ5 expression could be confirmed at protein level in myeloid and lymphoid cancer cell lines. (PDF 599 kb)
