# Supplementary material for: RECQ helicases are deregulated in hematological malignancies in association with a prognostic value
Source: Biomark Res. 2016 Feb 13;4:3. doi: 10.1186/s40364-016-0057-4 (PMC4752763; doi:10.1186/s40364-016-0057-4)
Supplement: Additional file 1: Figure S1. — RECQ helicase gene and protein expression in myeloid and lymphoid cell lines using using the human protein atlas database. RECQ1, RECQ4 and RECQ5 expression could be confirmed at protein level in myeloid and lymphoid cancer cell lines. (PDF 599 kb) [file 40364_2016_57_MOESM1_ESM.pdf]

RECQ1 expression

CELL LINE ATLAS ? »

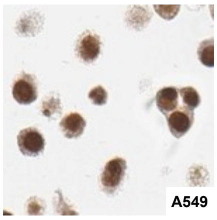

**Gene description**	RecQ helicase-like
**RNA expression**	Transcript detected at medium/high level in 38 cell lines
**Protein expression**	Protein detected at medium/high level in 45 cell lines
**Protein class**	Enzymes, Plasma proteins, Predicted intracellular proteins
**Reliability**	Supportive

**RNA** RNA Expression (FPKM) 200 100 0	Cell line	Cell line summary	**Protein** Antibody staining (score) 0 3000 6000
		myeloid cell lines	
	HEL	Erythroleukemia cell line	
	HL-60	Acute promyelocytic leukemia cell line	
	HMC-1	Mast cell leukemia cell line	
	K-562	Chronic myeloid leukemia cell line	
	NB-4	Acute promyelocytic leukemia cell line	
	THP-1	Acute monocytic leukemia cell line	
	U-937	Monocytic lymphoma cell line	
		lymphoid cell lines	
	Daudi	Human Burkitt lymphoma cell line	
	HDLM-2	Hodgkin lymphoma cell line	
	Karpas-707	Multiple myeloma cell line	
N/A	LP-1	Multiple myeloma cell line	
	MOLT-4	Acute lymphoblastic leukemia cell line	
	REH	Pre-B cell leukemia cell line	
	RPMI-8226	Multiple myeloma cell line	
	U-266/70	Multiple myeloma cell line	
	U-266/84	Multiple myeloma cell line	
	U-698	B-cell lymphoma cell line	

BLM expression

CELL LINE ATLAS ? »

**Gene description**	Bloom syndrome, RecQ helicase-like
**RNA expression**	Transcript detected at only low levels
**Protein expression**	Pending cell analysis
**Protein class**	Cancer-related genes, Disease related genes, Enzymes, Plasma proteins, Potential drug targets, Predicted intracellular proteins

**RNA** RNA Expression (FPKM) 20 10 0	Cell line	Cell line summary	**Protein** Antibody staining (score) 0 500 1000
		myeloid cell lines	
	HEL	Erythroleukemia cell line	N/A
	HL-60	Acute promyelocytic leukemia cell line	N/A
	HMC-1	Mast cell leukemia cell line	N/A
	K-562	Chronic myeloid leukemia cell line	N/A
	NB-4	Acute promyelocytic leukemia cell line	N/A
	THP-1	Acute monocytic leukemia cell line	N/A
	U-937	Monocytic lymphoma cell line	N/A
		lymphoid cell lines	
	Daudi	Human Burkitt lymphoma cell line	N/A
	HDLM-2	Hodgkin lymphoma cell line	N/A
	Karpas-707	Multiple myeloma cell line	N/A
N/A	LP-1	Multiple myeloma cell line	N/A
	MOLT-4	Acute lymphoblastic leukemia cell line	N/A
	REH	Pre-B cell leukemia cell line	N/A
	RPMI-8226	Multiple myeloma cell line	N/A
	U-266/70	Multiple myeloma cell line	N/A
	U-266/84	Multiple myeloma cell line	N/A
	U-698	B-cell lymphoma cell line	N/A

WRN expression

CELL LINE ATLAS ? »

**Gene description**	Werner syndrome, RecQ helicase-like
**RNA expression**	Transcript detected at medium/high level in 1 cell lines
**Protein expression**	Pending cell analysis
**Protein class**	Cancer-related genes, Disease related genes, Enzymes, Plasma proteins, Potential drug targets, Predicted intracellular proteins

RNA<br>RNA Expression (FPKM)<br>100 50 0	Cell line	Cell line summary	Protein<br>Antibody staining (score)<br>0 500 1000
		myeloid cell lines	
	HEL	Erythroleukemia cell line	N/A
	HL-60	Acute promyelocytic leukemia cell line	N/A
	HMC-1	Mast cell leukemia cell line	N/A
	K-562	Chronic myeloid leukemia cell line	N/A
	NB-4	Acute promyelocytic leukemia cell line	N/A
	THP-1	Acute monocytic leukemia cell line	N/A
	U-937	Monocytic lymphoma cell line	N/A
		lymphoid cell lines	
	Daudi	Human Burkitt lymphoma cell line	N/A
	HDLM-2	Hodgkin lymphoma cell line	N/A
	Karpas-707	Multiple myeloma cell line	N/A
N/A	LP-1	Multiple myeloma cell line	N/A
	MOLT-4	Acute lymphoblastic leukemia cell line	N/A
	REH	Pre-B cell leukemia cell line	N/A
	RPMI-8226	Multiple myeloma cell line	N/A
	U-266/70	Multiple myeloma cell line	N/A
	U-266/84	Multiple myeloma cell line	N/A
	U-698	B-cell lymphoma cell line	N/A

RECQ4 expression

CELL LINE ATLAS ? »

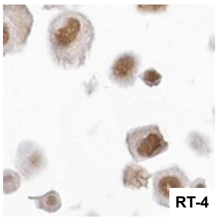

**Gene description**	RecQ protein-like 4
**RNA expression**	Transcript detected at medium/high level in 22 cell lines
**Protein expression**	Protein detected at medium/high level in 38 cell lines
**Protein class**	Cancer-related genes, Predicted intracellular proteins
**Reliability**	Uncertain

RNA<br>RNA Expression (FPKM)<br>100 50 0	Cell line	Cell line summary	Protein<br>Antibody staining (score)<br>0 2000 4000
		myeloid cell lines	
	HEL	Erythroleukemia cell line	
	HL-60	Acute promyelocytic leukemia cell line	
	HMC-1	Mast cell leukemia cell line	
	K-562	Chronic myeloid leukemia cell line	
	NB-4	Acute promyelocytic leukemia cell line	
	THP-1	Acute monocytic leukemia cell line	
	U-937	Monocytic lymphoma cell line	
		lymphoid cell lines	
	Daudi	Human Burkitt lymphoma cell line	
	HDLM-2	Hodgkin lymphoma cell line	
	Karpas-707	Multiple myeloma cell line	
N/A	LP-1	Multiple myeloma cell line	
	MOLT-4	Acute lymphoblastic leukemia cell line	
	REH	Pre-B cell leukemia cell line	
	RPMI-8226	Multiple myeloma cell line	
	U-266/70	Multiple myeloma cell line	
	U-266/84	Multiple myeloma cell line	
	U-698	B-cell lymphoma cell line	

RECQ5 expression

CELL LINE ATLAS ? »

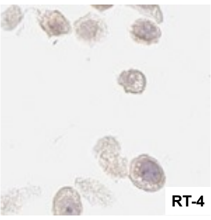
RT-4

**Gene description**	RecQ protein-like 5
**RNA expression**	Transcript detected at medium/high level in 1 cell lines
**Protein expression**	Protein detected at only low levels
**Protein class**	Enzymes, Plasma proteins, Predicted intracellular proteins
**Reliability**	Supportive

**RNA**<br>RNA Expression (FPKM)<br>20 10 0	Cell line	Cell line summary	**Protein**<br>Antibody staining (score)<br>0 500 1000
		myeloid cell lines	
	HEL	Erythroleukemia cell line	
	HL-60	Acute promyelocytic leukemia cell line	
	HMC-1	Mast cell leukemia cell line	
	K-562	Chronic myeloid leukemia cell line	
	NB-4	Acute promyelocytic leukemia cell line	
	THP-1	Acute monocytic leukemia cell line	
	U-937	Monocytic lymphoma cell line	
		lymphoid cell lines	
	Daudi	Human Burkitt lymphoma cell line	
	HDLM-2	Hodgkin lymphoma cell line	
	Karpas-707	Multiple myeloma cell line	
N/A	LP-1	Multiple myeloma cell line	
	MOLT-4	Acute lymphoblastic leukemia cell line	
	REH	Pre-B cell leukemia cell line	
	RPMI-8226	Multiple myeloma cell line	
	U-266/70	Multiple myeloma cell line	
	U-266/84	Multiple myeloma cell line	
	U-698	B-cell lymphoma cell line	
